# Investigation of the Virome and Characterization of Issyk-Kul Virus from Swedish *Myotis brandtii* Bats

**DOI:** 10.3390/pathogens12010012

**Published:** 2022-12-21

**Authors:** Harindranath Cholleti, Johnny de Jong, Anne-Lie Blomström, Mikael Berg

**Affiliations:** 1 Section of Virology, Department of Biomedical Sciences and Veterinary Public Health, Swedish University of Agricultural Sciences (SLU), P.O. Box 7028, 750 07 Uppsala, Sweden; 2 Swedish Biodiversity Centre (CBM), SLU, P.O. Box 7016, 750 07 Uppsala, Sweden

**Keywords:** metagenomics, virome, *Myotis brandtii*, bats, Issyk-Kul virus, Sweden

## Abstract

Bats are reservoirs for many different viruses, including some that can be transmitted to and cause disease in humans and/or animals. However, less is known about the bat-borne viruses circulating in Northern European countries such as in Sweden. In this study, saliva from *Myotis brandtii* bats, collected from south-central Sweden, was analyzed for viruses. The metagenomic analysis identified viral sequences belonging to different viral families, including, e.g., *Nairoviridae*, *Retroviridae*, *Poxviridae*, *Herpesviridae* and *Siphoviridae*. Interestingly, through the data analysis, the near-complete genome of Issyk-Kul virus (ISKV), a zoonotic virus within the *Nairoviridae* family, was obtained, showing 95–99% protein sequence identity to previously described ISKVs. This virus is believed to infect humans via an intermediate tick host or through contact with bat excrete. ISKV has previously been found in bats in Europe, but not previously in the Nordic region. In addition, near full-length genomes of two novel viruses belonging to *Picornavirales* order and *Tymoviridae* family were characterized. Taken together, our study has not only identified novel viruses, but also the presence of a zoonotic virus not previously known to circulate in this region. Thus, the results from these types of studies can help us to better understand the diversity of viruses circulating in bat populations, as well as identify viruses with zoonotic potential that could possibly be transmitted to humans.

## 1. Introduction

Bats (*Chiroptera)* are reservoirs for a large number of different viruses, including zoonotic viruses, some of them known to cause diseases in humans and/or animals, such as Hendra virus, Nipah virus, Ebola virus, Marburg virus and SARS virus [1,2,3,4,5,6,7]. The transmission of bat-borne viruses to humans often requires an intermediate host; for example, SARS-CoV-1 and MERS-CoV originated from bats and transmitted to humans through civets and dromedary, respectively [8], although direct spill-over from bat to human is also known to occur for certain bat-borne viruses [9]. Despite the impact of the bat-borne zoonotic diseases [10], viruses in bats have historically been poorly characterized. However, in recent years, the advancement of high-throughput sequencing (HTS) combined with metagenomic analyses has led to an increased number of studies characterizing the virome of different bat species from different locations. Overall, these studies show that the viral composition can vary based on both geographic locations as well as on bat species, and they shed light on the great viral diversity found in bats [11,12,13].

A majority of studies investigating the presence of bat-borne viruses have been conducted in Asia, Africa and in the Americas, although in recent years increasing data from Europe are becoming available. However, only a small number of zoonotic bat-borne viruses are known to circulate in Europe, for example, bat lyssaviruses and mammalian orthoreoviruses [14,15,16,17]. In addition, Issyk-Kul virus (ISKV), belonging to the *Bunyavirales* order, was recently detected in German bats. The viral ISKV strain identified in Germany showed close similarity to a pathogenic ISKV strain (LEIV315K) previously identified in Central Asia [18]. ISKV is known to cause sporadic febrile outbreaks in humans with symptoms such as headache, myalgia and nausea; however, no fatal cases have been reported [19].

In Sweden, 19 bat species have been officially recognized [20] and very little is known about the viral diversity in these bats. However, a few studies have been carried out. For example, *Myotis daubentonii* bats from the southern part of Sweden have been shown to carry antibodies for European bat lyssavirus [21] and, through a metagenomic study, been shown to carry alphacoronavirus [22]. In addition, we have recently characterized the virome from feces of *Pipistrellus pygmaeus* bats collected in the Uppland region of Sweden [23]. In that study, three near-full length genomes of bat coronaviruses and two picornaviruses were characterized. Thus, in this study we wanted to further expand the knowledge of which viruses are circulating in the Swedish bat populations. As in our previously mentioned study, we therefore collected bat samples in the Uppland region but focusing on another bat species (*Myotis brandtii)* also known to be present in this region. Saliva was used as it is known that viruses have spilled over from bats to different mammals through this route.

## 2. Materials and Methods

### 2.1. Ethics Statement

The ethical permit to conduct this study was approved by the Swedish Board of Agriculture (5.2.18-02469/2020) and the ethical committee of Uppsala district court (5.8.18-0713/2020).

### 2.2. Bat Saliva Sampling

*Myotis brandtii (M. brandtii)* bats were sampled from a roost in south-central Sweden, approximately 13 km east of Östhammar (Sundsveden, 60°15′08”N, 18°36′26”E) and close to the Baltic sea. All the bats (*n* = 11) were trapped using mist nets positioned outside the roost. Species were morphologically identified on site. Prior to release, saliva swabs (*n* = 11) were collected and preserved on ice in 500 μL of PBS solution. The samples were transported to the laboratory and stored at −80 °C until further use.

### 2.3. RNA Processing, Sequencing and Bioinformatic Analysis

The individual samples were processed, sequenced and bioinformatically analyzed as previously described by Cholleti et al. [23]. Briefly, RNA was extracted from 200 µL swab solution using a combination of TRIzol^TM^ LS reagent (Invitrogen, Carlsbad, CA, USA) and Genejet RNA extraction kit (Thermo Fisher Scientific, Waltham, MA, USA). In total, 6 to 10 μL of RNA from each bat was pooled into 4 pools (up to 3 samples/pool), followed by DNase treatment using an RNAs-free DNase set (Qiagen, Hilden, Germany) and ribosomal RNA depletion using a RiboMinus Eukaryotic System v2 (Thermo Fisher Scientific, Waltham, MA, USA) to remove the host and bacterial genetic material. The remaining RNA was amplified using the Ovation RNA-seqV2 system (Tecan, Männedorf, Switzerland). The quantity of ovation amplicons was determined using the 4200 Tapestation System (Agilent Technologies, Santa Clara, CA, USA). The amplicons were further pooled into two pools before library preparation and sequencing, which were carried out by the SNP&SEQ Technology platform in Uppsala, Sweden. The Illumina-generated sequences from the two pools were merged into a single dataset and analyzed with different bioinformatic tools. The adapters were trimmed, and low-quality sequences and duplicates were removed. The good-quality sequences were submitted to Kaiju web server (NCBI BLAST nr+euk database) to assign a taxon to each sequence and assembled using a de novo assembler, Megahit [24]. The assembled contigs were then taxonomically classified by BLASTx using Diamond [25] and the output files were visualized by MEGAN [26]. The good-quality reads were mapped back to near full-length genomes for base correction and the consensus sequences were exported. The ORFs and UTRs of each segment were determined using the closest reference genome and the ORF finder tool from the NCBI. The ORFs were further analyzed for conserved amino acid motifs using HHpred [27] against Pfam [28] and PDB [29] databases.

### 2.4. Sanger Sequencing of Missing Genomic Regions

Specific viral PCR primers were designed using the contig information and the closest reference genomes to confirm the presence of the virus in individual bats and to sequence gaps in the genome, including UTRs. The total RNA was converted to cDNA by incubating RNA with dNTPs and random primers at 65 °C for 5 min, and with SuperScript III reverse transcriptase (Invitrogen, Carlsbad, CA, USA) at 25 °C for 5 min, 50 °C for 60 min and 70 °C for 15 min. PCR amplification was performed using AmpliTag Gold^TM^ DNA Polymerase (Applied Biosystems, Foster City, CA, USA) and thermal cycling was initiated with a denaturation step at 95 °C for 10 min, followed by 40 cycles of 95 °C for 30 s, 57–60 °C for 30 s, 72 °C for 1 min and a final extension at 72 °C for 7 min. The amplified products were purified with a GeneJet PCR purification kit (Thermo Fisher Scientific, Waltham, MA, USA) and sequenced at Macrogen Europe (Macrogen Europe BV). The primers used in this study are listed in the supplementary Table S1.

### 2.5. Phylogenetic Analysis

Reference genomes from the *Orthonairovirus* genus, from different families of the *Picornavirales* order and from the *Tymoviridae* family were obtained from NCBI. The representative sequences from each group/genus/family were used as proposed by the International Committee on Taxonomy of Viruses. The nucleotide sequences were converted to amino acids and sequence alignments were generated using the MUSCLE plugin with the default settings and manually curated in MEGAx [30]. The phylogenetic trees were constructed using a maximum likelihood model by IQ-TREE v1.6.9 [31]. The best fitting substitution model was determined based on Bayesian Information Criterion by the ModelFinder functionality [32] in IQ-TREE, and the reliabilities of trees were evaluated with the ufbootstrap procedure with 1000 replicates. Trees were visualized using FigTree v1.4.4 (http://tree.bio.ed.ac.uk/software/figtree/, accessed on 23 October 2022). Bootstrap values greater than 50 are displayed at nodes.

## 3. Results

During July 2020, a total of 11 saliva samples were collected from bats trapped from a house roost located near the Baltic sea in Sundsveden in Uppsala County, Sweden. All the bats were determined to be females, one year old and belonging to the *M. brandtii* (Eversmann, 1845) species. A total of 179,890,909 paired-end (PE) reads were generated from the Illumina NovoSeq6000 sequencer. After removing the low-quality reads and exact duplicate reads (23%), 138,889,457 PE reads were obtained and used for further analysis (Table S2.) The metagenomic classification of the filtered reads was done using the Kaiju web server. Approximately 58% of the filtered PE reads could be classified and the majority of them belonged to bacteria (97.8%) and eukaryota (2.13%). The reads classified as viruses and archaea accounted for 0.019% (27,495 PE reads) and 0.004% (3,518 PE reads) of the total filtered reads, respectively.

### 3.1. Taxonomic Classification of Viral Reads from Bat Saliva

**Figure 1:**
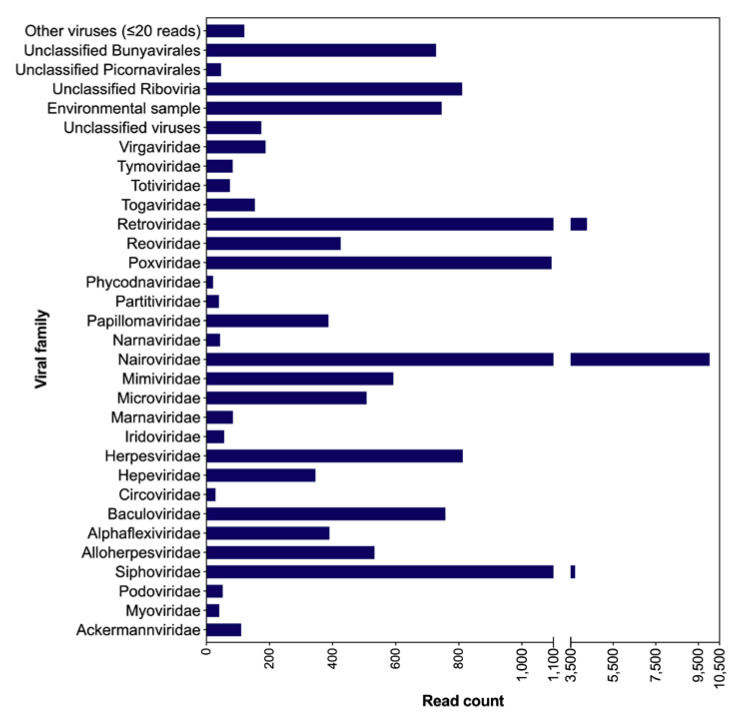
Taxonomic classification of viral reads at the family level, at the protein level. Scale breaks are introduced in the x-axis for the visualization of the variable number of viral counts. The exact numbers of viral read counts are listed in Table S3.

The classification of the reads identified sequences related to 47 viral families in the saliva of the bats; however, for a number of these families, only a few viral reads were classified. The viral families identified include, e.g., *Nairoviridae*, *Retroviridae*, *Poxviridae*, *Herpesviridae*, *Baculoviridae*, *Mimiviridae* and phages (*Ackermannviridae*, *Podoviridae* and *Siphoviridae*) (Figure 1). Noticeably, a large number of viral reads could not be classified at the family level. The exact viral read counts for each viral family/group are provided in Table S3. The assembly of filtered reads resulted in 174 contigs related to viruses, a few of them representing near full-length and partial viral genomes, which were further characterized.

### 3.2. Nairoviridae Family

A total of 10,042 reads were classified as *Nairoviridae*, as were six contigs. The contigs showed the closest protein identity (83–100%) with the nucleoprotein (S segment), the envelop glycoprotein (M segment) and the RNA-dependent RNA polymerase (RdRp; L segment) of Issyk-Kul virus (ISKV), an Orthonairovirus. Together, these contigs covered nearly the full length or partial genomic segments (Table S4), and gaps were filled by PCR and Sanger sequencing. Thereafter, all the filtered reads were re-mapped to confirm the sequence of the ISKV genome and we determined complete coding regions for all three segments (S, M and L).

#### Genome Characteristics of ISKV

**Figure 2:**
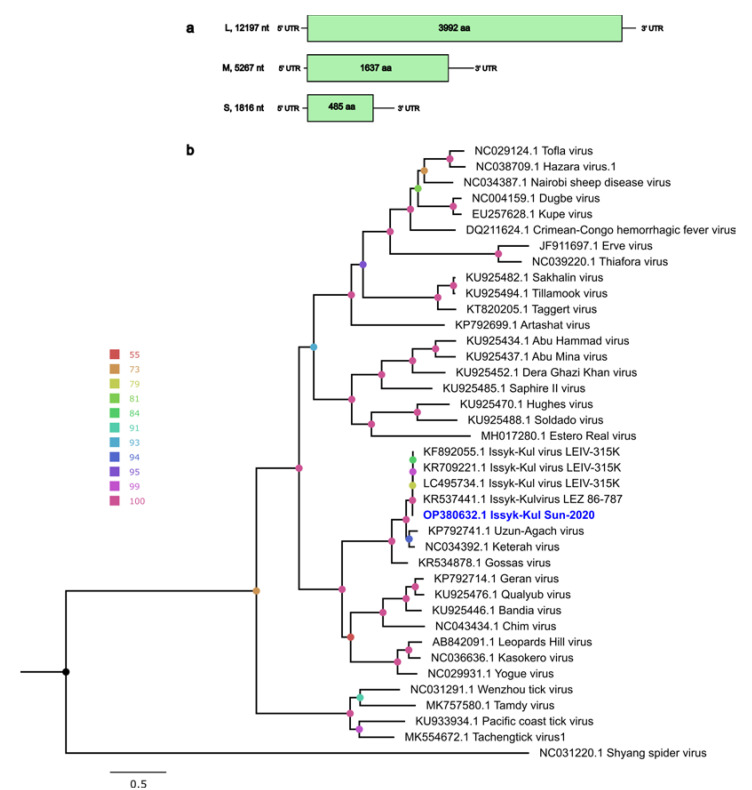
(**a**) The genome organization and nt length for each S, M and L segment with nt and ORF lengths. (**b**) Maximum-likelihood phylogenetic tree based on the full-length L protein from representative orthonairoviruses and Issyk-Kul viruses. The ISKV from this study is shown in blue. The color of node circles indicates the corresponding ultrafast bootstrap support values generated by IQ-TREE. The Shyang spider virus was used as an outgroup.

ISKV has a tripartite negative-sense RNA genome. The lengths of the S, M and L segments from this study were determined as 1,816, 5,267 and 12,197 nt, respectively (Figure 2a). The organization of the Swedish ISKV genome (isolate Sun-2020) is consistent with previously characterized ISKVs and other members in the genus *Orthonairovirus*, each containing a single ORF encoding the nucleocapsid protein (S segment, 485 aa), glycoprotein (M segment, 1,637 aa) and RdRp (L segment, 3,992 aa). The S segment is flanked by a 44 nt 5′ UTR and 314 nt 3′ UTR. The M segment is flanked by a 14 nt 5′ UTR and 339 nt of 3′ UTR, while the L segment consists of 43 nt of 5′ UTR and 175 nt of 3′ UTR. The conserved ISKV terminal sequence, a typical feature of the genus *Orthonairovirus*, with 5′- UCUCAAAGA, was identified in all three segments. Unfortunately, we failed to sequence the complete 3’ UTR and could thus not obtain the expected reverse-complementary 3′-UCUUUGAGA sequence which is a typical feature of the genus *Orthonairovirus*. A BLASTx search of the segments showed 95–99% identity to other ISKVs from Germany and Kyrgyzstan, isolated from *Carios vespertilionis* ticks and suckling mice (originally isolated from *Nyctalus noctula* bats) (Table 1). The genomic sequences were deposited in GenBank with the accession numbers OP380630–OP380632.

Further, an ML phylogenetic analysis of the N protein (S segment), glycoprotein (M segment) and RdRp (L segment) showed that our sequences closely clustered with other ISKVs and formed a well-supported monophyletic clade with viruses in the Keterah group (Figure 2b, Figures S1 and S2). The eleven individual bats were screened with PCR primers targeting a 400 bp-long region of the L segment, and this showed that only one bat was positive for ISKV.

### 3.3. Other Viral Genomes

**Figure 3:**
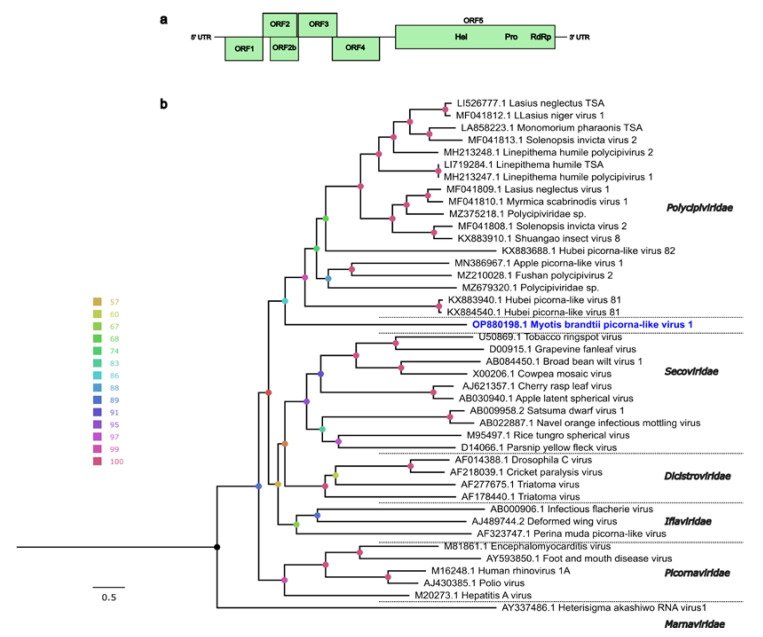
(**a**) Genomic organization and predicted ORFs of the MbplV-1 genome. The genome is represented by a black line and ORFs are indicated in light green color. The putative conserved domains are shown in the ORF boxes and were predicted by HHpred using Pfam and PDB databases: Hel, helicase; Pro, protease; RdRp, RNA-dependent RNA polymerase. (**b**) Maximum-likelihood phylogenetic tree based on the full-length ORF5 protein of representative viruses from different families in *Picornavirales* order and MbplV-1 (in blue). The color of the node circles indicates corresponding ultrafast bootstrap support values generated by IQ-TREE. The Heterisigma akashiwo RNA virus 1 from the *Marnaviridae* family was used as an outgroup.

Apart from the ISKV, the viral contigs showed similarities to different viruses from bats, insects and plants. For example, one long contig (11,121 nt) was classified to the order *Picornavirales*. This monopartite-positive and single-stranded RNA genome was predicted to have five different ORFs, four in its 5′ (ORF1–4) and a single long ORF in its 3′ (ORF5) (Figure 3a). The genomic positions for each ORF and UTR are shown in the supplementary Table S5. The BLAST protein search for ORF1–4 yielded no matches in the NCBI-nr database; however, a partial protein sequence of ORF5 matched with the RdRp of an unclassified virus in the *Polycipiviridae* family (*Polycipiviridae* sp., GenBank accession no. MZ375218.1), with a protein identity of 24%, suggesting the identification of a highly divergent virus. Mapping the sequence reads back to this viral contig showed a high coverage for each base across the genome. Further, the functions for the coding proteins were predicted with HHpred using the Pfam and PDB databases. The ORFs 1, 3 and 4 were predicted to encode picornavirus-like capsid proteins and ORF5 was predicted to encode a helicase, a protease, and a RdRp (Figure 3a). This genome structure is consistent with other viruses in the *Polycipiviridae* family. However, ORF2 and a putative overlapped ORF had no significant matches with HHpred. The phylogenetic tree was generated using the full-length ORF5 amino acid sequence and representative members in the order *Picornavirales* (Figure 3b), and this placed the virus from this study on a separate monophyletic clade with the group members of the *Polycipiviridae* family. Considering the genome organization, amino acid sequence identity to previously known viruses and predicted protein domains, we propose a new virus, Myotis brandtii picorna-like virus 1 (MbplV-1, GenBank accession no. OP880198), tentatively placed in the order Picornavirales. 

**Figure 4:**
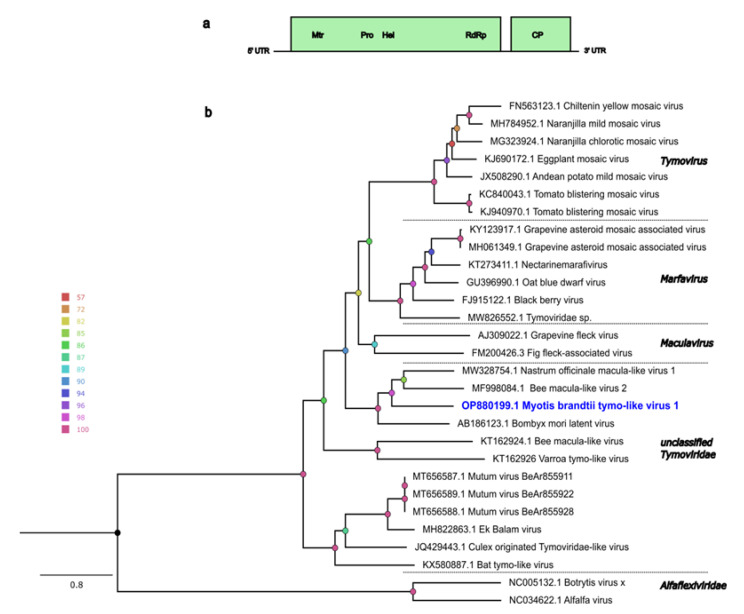
(**a**) Genomic organization of the MbTLV-1 genome characterized in this study. The ORF1 and ORF2 are indicated in light green and purple boxes, respectively. The putative conserved domains are shown in the ORF boxes and were predicted by HHpred using Pfam and PDB databases. Mtr, methyltranferase; Pro, papain-like protease; Hel, helicase; RdRp, RNA-dependent RNA polymerase; CP, coat protein. (**b**) Maximum-likelihood phylogenetic tree based on full-length ORF1 protein of representative viruses from *Tymoviridae* family and MbplV-1(in blue). The color of node circles indicates corresponding ultrafast bootstrap support values generated by IQ-TREE. Viruses from *Alfaflexiviridae* were used as an outgroup.

Another long viral contig (6162 nt) belonging to the *Tymoviridae* viral family was genetically characterized. The ORF analysis shows that the genome consists of two complete ORFs: ORF1 and ORF2 (Figure 4a). The longest ORF (ORF1) showed the highest BLASTp identity (58%) with a replicase protein of *Nasturtium officinale* macula-like virus 1 (GenBank accession no. MW328754.1), and functional domains for methyltransferase, cysteine protease, an RNA helicase, and an RdRp were identified through the HHpred search. ORF2 showed the highest identity (47%) with the coat protein of the same virus. The genome organization of ORF1 and ORF2 is consistent with what is seen for other viruses in the *Tymoviridae* family. Phylogenetic analysis using the full-length ORF1 showed that this genome clustered with unclassified tymoviruses and most closely with *Nasturtium officinale* macula-like virus 1 and Bee macula-like virus 2 (Figure 4b). As per the demarcation criteria defined by ICTV for the *Tymoviridae* family (the overall sequence identity of the characterized genome should be less than 80% and the Capsid protein identity less than 90%), this genome is proposed to be a novel virus, tentatively named *Myotis brandtii* tymo-like virus 1 (MbTLV-1, GenBank accession no. OP880199), and assigned to the *Tymoviridae* family. However, the genus is yet to be determined. The genomic positions for each ORF and UTRs are shown in supplementary Table S6.

The majority of the other viral contigs showed similarities to unclassified RNA viruses, many of which were previously identified in different insect species from China [33]. A number of contigs related to phages, e.g., *Siphoviridae*, *Myoviridae* and *Cuadovirales*, were identified. Nine reoviral contigs (273–949 nt) were assembled and these showed protein identity (40–99%) to Kadipora virus, Cimodoviruses, orbivirses and different cypoviruses. Contigs related to *Microviridae*, *Retroviridae*, *Alfaflexiviridae*, unclassified Riboviria and unclassified viruses were also identified. The list of other contigs and their identities are shown in the supplementary Table S7.

## 4. Discussion

*Myotis brandtii* (Vespertillionidae) is distributed throughout Europe and parts of western Asia. In this study, we used high-throughput sequencing to determine the viral composition of *Myotis brandtii* captured in Uppland, Sweden. However, only a small proportion of the classified reads were assigned to the virus group while the majority were bacterial reads. Considering this, we believe the inclusion of a filtration step that filtrates the saliva through a 0.22 μM or a 0.45 μM filter prior to RNA extraction would be beneficial to increase the proportion of viral reads. Despite this, we show that a number of different viral genomes could be found in the saliva of *M.brandtii* bats collected from the same roost. For example, viral families such as *Siphoviridae*, *Hepeviridae*, *Herpesviridae*, *Nairoviridae*, *Poxviridae*, *Reoviridae*, *Retroviridae* and *Alloherepsviridae* were identified. Some of these viral families have members with zoonotic potential that have been previously reported in European bats [34]. Similar viral families have been detected in other bat metagenomic studies from, e.g., Germany [11], South Africa [35], Denmark [36], Argentina [37] and Croatia [38]. However, the comparison of viral diversity between different studies is complex and may be attributed to different factors, for example, bat species, sampling location, season, year, sample size, sample type, sample preparation and sequence analysis [38,39,40,41]. This is further highlighted by the fact that the viruses from this study that were genetically characterized were not detected in our previous metagenomic study investigating *Pipistrellus pygmaeus* bats in Uppland [23]. Furthermore, in the *Pipistrellus pygmaeus* bats, a majority of the viral reads were coronaviruses, while in these *M. brandtii*, no coronaviruses were detected. However, coronavirus was detected in *Myotis daubentoniid* from southern Sweden.

In this study, six contigs were classified as ISKV, a *Orthonairovirus*, and these showed an identity of 83–99% at the amino acid level with previously described ISKV genomes. Phylogenetic analysis of each segment shows that this new strain clustered with known ISKVs in the Keterah group within the genus *Orthonairovirus*, family *Nairoviridae*. This Swedish strain has been named “Sun-2020”, indicating its origin (Sundsveden) and the year of sample collection (2020). ISKV was first isolated in 1970 from a *Nyctalus noctula* bat and from the argasid tick *Carios vespertillions* (formerly *Argas vespertillions*) in Kyrgyzstan [42], and was eventually also detected in Tajikistan, Kazakhstan and Germany [18,43,44]. ISKVs have also been isolated from different kinds of species, including humans, bats, ticks, mosquitoes and flies [19,45,46], indicating their broad host range and zoonotic capacity. ISKV has also been identified in the hard tick *Ixodes vespertillions* [19], and the main hosts of these two mentioned ticks are different species of bats [47,48,49], suggesting the circulation of the virus between bats and these ticks. The first-time detection of this virus in Germany [18] was in *Eptesicus nilsonii* bats, which are bats commonly found throughout Asia and Europe. In Sweden, similar to *M. branditii* bats, they roost in houses. It is speculated that ISKV can be transmitted by tick bites and human exposure to bat feces and urine [19], and based on the results from our study, saliva from bats could also be a potential exposure route to humans and/or animals. However, further studies are needed to, e.g., determine the presence of infectious ISKV in the saliva in order to prove whether ISKV could spill over to other species through the saliva. Such studies would also benefit from a larger sample set. Several viruses in the *Nairoviridae* family are transmitted through ticks, some of which have the ability to cause severe diseases in humans and animals, e.g., Crimean-Congo hemorrhagic fever virus [50]. In addition, there are several other members of the *Orthonairovirus* genus that can cause non-lethal disease in humans, such as Dugbe virus [51], Nairobi sheep disease virus [52] and Erve virus [53]. ISKV was previously detected in different organs of bats, such as the liver, spleen and lung tissue, suggesting systemic infection [18]. A recent study showed that ISKV can replicate in different mammalian and tick-derived cell lines, and is also capable of causing severe liver lesions and death in type I interferon receptor knockout mice [54]. In our study, only one out of eleven sampled bats were positive for ISKV in the saliva, and further studies are needed to determine, e.g., the prevalence of this virus in the bat population, the potential viral fluctuation and if virus could be isolated from these bats.

Several studies have demonstrated that bats harbor a wide range of viruses belonging to *Picornaviridae*, *Iflaviridae*, *Dicistroviridae*, *Secoviridae* and *Polycipiviridae* families in the order *Picornavirales*, and these studies have suggested that these viruses originate mainly from the arthropods eaten by bats [38,39,55,56]. In this study, we identified a highly divergent viral genome, MbplV-1, that was classified in the *Picornavirales* order and that showed only very low amino acid similarity to *Polycipiviridae* sp. The analysis of the genomic structures (ORF number, putative functions and orientation) supports the placement of this virus in the order *Picornavirales*. The genome organization of MbplV-1 is similar (with an overlapping putative ORF2b, Figure 3a) to polycipiviruses, as described by Olendraite et al. [57]. The phylogenetic analysis placed MbplV-1 with viruses in the *Polycipiviridae* family, although on a separate branch. Whether Mbp1V-1 belongs to the *Polycipiviridae* family or will end up in a separate family is yet to be determined. Viruses in the *Polycipiviridae* family contain positive-sense, single-stranded RNA viruses and consist of the genera *Sopolycivirus* (ant-specific viruses), *Hupolycivirus* (crustacean- and insect-associated viruses) and *Chipolycivirus* (arachnid-associated viruses). MbplV-1 could have originated from the insect diet of Myotis bats; however, the host adaptation and range of this virus needs to be evaluated.

Bats have also been shown to harbor viruses related to the *Tymoviridae* family, and these have also been linked to the bat feed [11,23,38]. Members of this family consist of positive-sense, single-stranded RNA with a genome size ranging from 6 to 7.5 kb, and they have one to four ORFs depending on the genus. In this study, a novel virus, MbTLV-1, related to the *Tymoviridae* family, was characterized. The genome showed low protein identity (48–58%) to previously described tymoviruses, but the genome structure and conserved protein domains for Mtr, Pro, Hel, RdRp and coat protein were shown to be consistent with other viruses from this family. Phylogeny analysis showed that MbTLV-1 clustered with unclassified *Tymoviridae* viruses. The genome identified in this study could possibly be connected to the diet, as dicotyledonous plants are the primary hosts of these viruses, and with some exceptions they are transmitted by beetles, leafhoppers and mosquitoes [58,59].

Overall, the virome analysis shows that the investigated *M. brandtii* bats harbor a great number of viruses. Importantly, ISKV, a zoonotic orthonairovirus, was reported for the first time in the Nordic region. The finding of this virus in the saliva of bats could indicate a possible spill-over route to humans. In addition, genomes of highly divergent viruses related to the *Picornavirales* order and to the *Tymoviridae* family were characterized. This study supports previous findings that bats are natural reservoirs for many different viruses, including viruses with zoonotic potential. The metagenomic analysis of viruses from bat saliva at the roost level represents an ideal non-invasive, high-throughput method to understand viral diversity and discover novel viruses.

## Figures and Tables

**Table 1:** The genomic segments of ISKV, their lengths, ORF positions, UTRs and closest identities at the nucleotide and protein level.

Segment	Length, nt	ORF Position	ORF Length, nt/aa	Length of 5’ UTR, nt	Length of 3’ UTR, nt	% of ID, nt Level ^1^	% of ID, aa Level ^2^	Closest Virus Name
S	1816	45–1502	1448/485	44	314	97.91	99.59	Issyk-Kul virus isolate LEZ 86-787 (KR537443)
M	5267	15–4828	4914/1637	14	339	93.07	95.17	Issyk-Kul virus isolate LEIV-315K (KR709220)
L	12197	44–12022	11979/3992	43	175	98.61	99.62	Issyk-Kul virus isolate LEZ 86-787 (KR537441)

^1^—nucleotide level; ^2^—amino acid level.

## Data Availability

The short paired-end raw sequencing data from this study have been deposited in SRA under the BioProject ID PRJNA877456, and the viral genomes in GenBank with the accession numbers OP380630-OP380632, OP880198 and OP880199.

## Supplementary Materials

The following supporting information can be downloaded at: https://www.mdpi.com/article/10.3390/pathogens12010012/s1, Table S1: Primers used in this study; Table S2: Sequencing data, quality check and classification; Table S3: Metagenomic classification of viral reads; Table S4: Contigs classified as *Nairoviridae* family; Table S5: *Myotis brandtii* picorna-like virus 1 ORF positions in the genome; Table S6: *Myotis brandtii* tymo-like virus 1 ORF positions in the genome; Table S7: Assembled contigs classified as viruses; Figure S1 and S2: ML phylogenetic tree based on glyco- and N protein of ISKV and other members in the *Nairoviridae* family.
