# Supplementary material for: Investigation of the Virome and Characterization of Issyk-Kul Virus from Swedish Myotis brandtii Bats
Source: Pathogens. 2022 Dec 21;12(1):12. doi: 10.3390/pathogens12010012 (PMC9861107; doi:10.3390/pathogens12010012)
Supplement: Supplementary file 1 [file pathogens-12-00012-s001.zip › Supplematary Figures.pdf]

pathogens

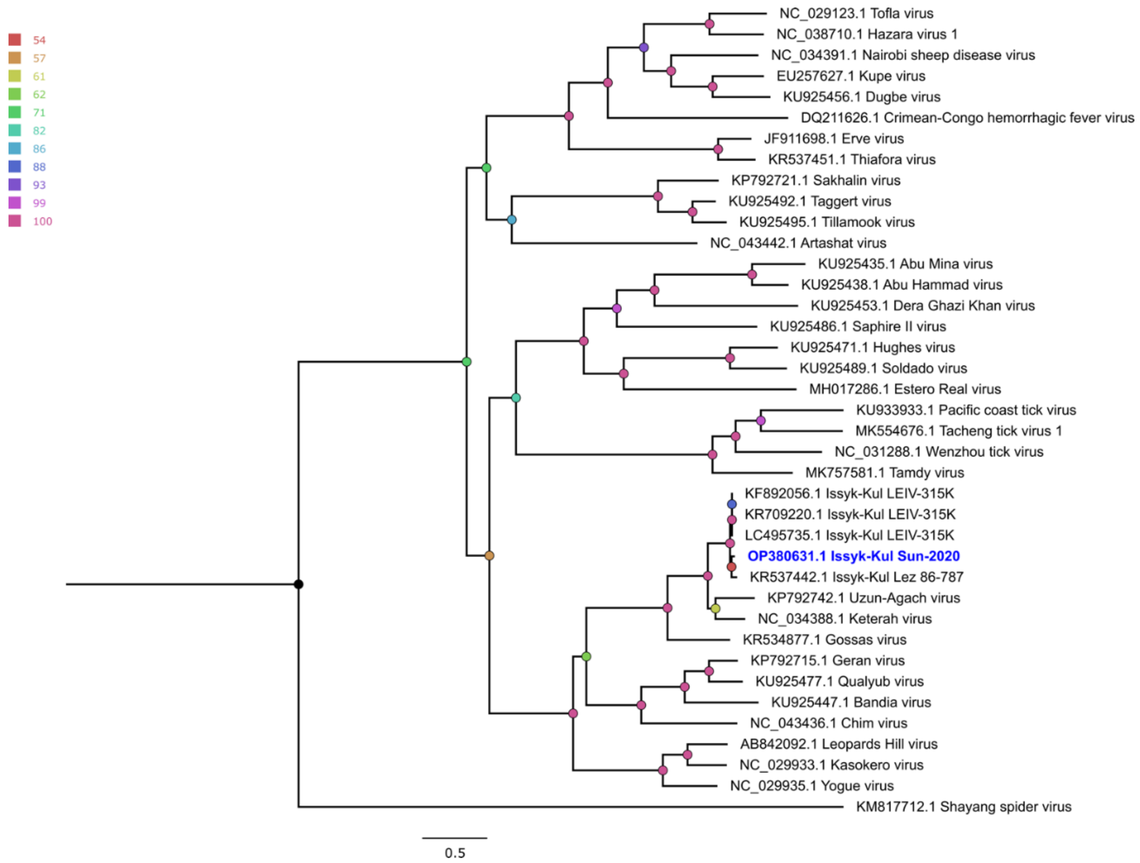

Figure S1: ML Phylogenetic tree based on the full-length glycoprotein from representative orthonairoviruses and Issyk-Kul viruses. The ISKV from this study is shown in blue. The color of node circles indicates the corresponding ultrafast bootstrap support values generated by IQ-TREE. The shyang spider virus was used as an outgroup.

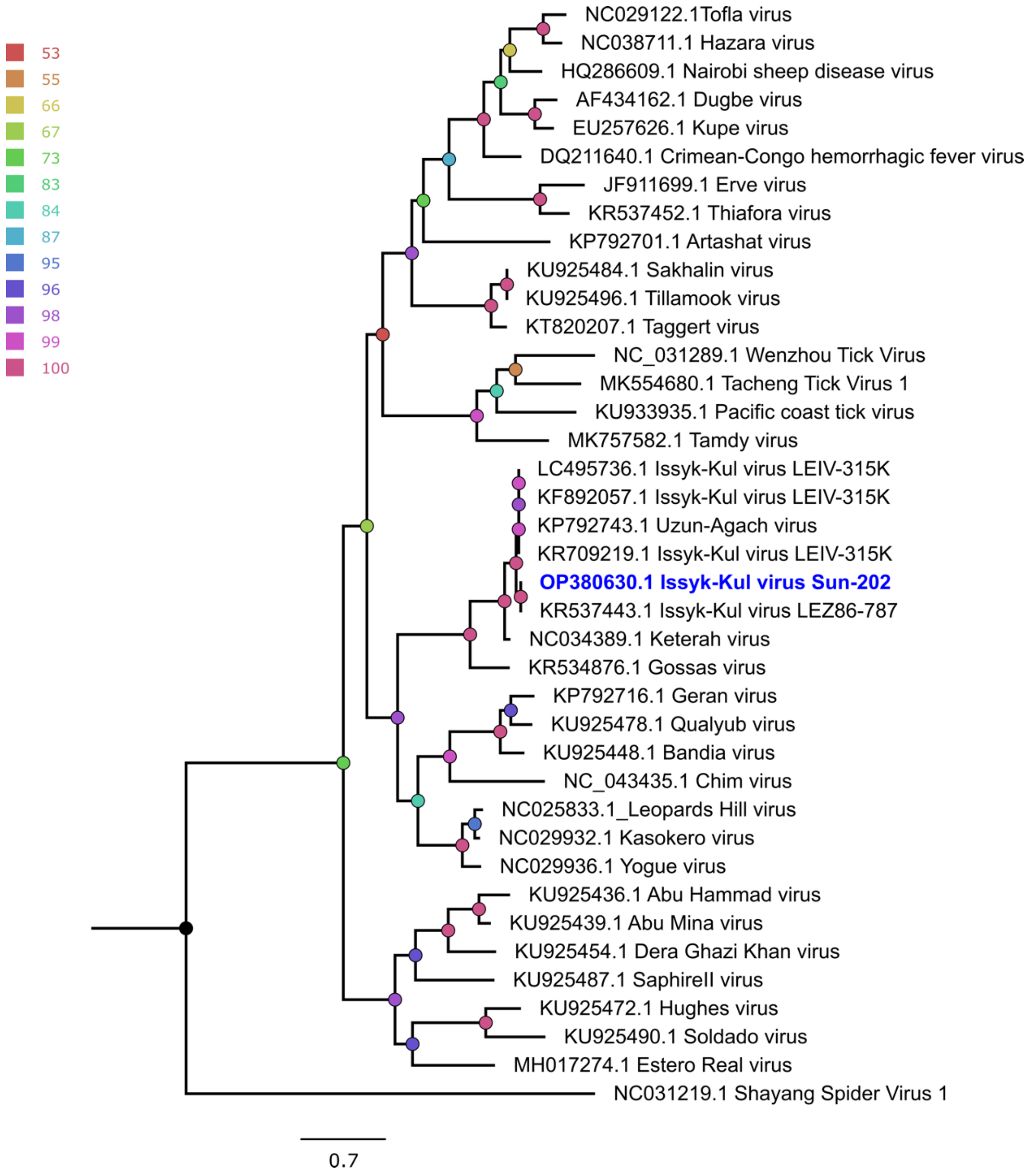

Figure S2: ML Phylogenetic tree based on the full-length N protein from representative orthonairoviruses and Issyk-Kul viruses. The ISKV from this study is shown in blue. The color of node circles indicates the corresponding ultrafast bootstrap support values generated by IQ-TREE. The shyang spider virus was used as an outgroup.
